# Promoting surveillance of healthcare related infections (HCRI) in Manhiça (Mozambique)

**DOI:** 10.1186/1753-6561-5-S6-O19

**Published:** 2011-06-29

**Authors:** A Trilla, J Vila, G Trilla, S Mayola, CISM/CRESIB

**Affiliations:** 1 CRESIB, Barcelona, Spain; 2 Hospital Clinic - University of Barcelona, Barcelona, Spain

## Introduction

The Centro de Investigação em Saúde de Manhiça (CISM) is a research institution whose mission is to promote and conduct biomedical research in priority health areas. The CISM is located in Manhiça, 80km from Maputo (Mozambique).

## Methods

The CISM has 3 well developed platforms (demographic, geographic and morbidity surveillance platforms) crucial. These platforms cover a study area of 500 km^2^ with close to 84,000 inhabitants. In this area, all houses are geo-positioned (GPS) and the population is under demographic surveillance. The morbidity surveillance system collects information on all paediatric outpatient visits and admissions to the Manhiça District Hospital. The CISM research agenda is directed at the priority health problems in Mozambique. The CISM maintains stable research collaborations with the HCB/UB and the Barcelona Centre for International Health Research (CRESIB). We are conducting a project to develop a prevalence survey system for HCRI, based on the Spanish EPINE study with more than 20 years of experience. Questionnaires as well as definitions will be tested in a pilot study at the CISM this summer, and data collected and analyzed following the standard protocol. The CISM contributes to the strengthening of human resources in the country through the training of researchers and other technical personnel. Physicians and Nurses from the CISM will be trained is the use of the surveillance tool, to ensure that the community benefits from research results.

## Conclusion

Improving healthcare provision in the Manhiça District is one of the CISM’s priorities. There is no data regarding the rates and types of HCRI in Mozambique, and simple, easy to do prevalence studies could be a way to approach this health problem.

## Disclosure of interest

None declared.

